# Membrane Protein Bcest Is Involved in Hyphal Growth, Virulence and Stress Tolerance of *Botrytis cinerea*

**DOI:** 10.3390/microorganisms11051225

**Published:** 2023-05-06

**Authors:** Wei Zhang, Bei-Bei Ge, Zhao-Yang Lv, Kyung Seok Park, Li-Ming Shi, Ke-Cheng Zhang

**Affiliations:** 1 State Key Laboratory of Biology of Plant Diseases and Insect Pests, Institute of Plant Protection, Chinese Academy of Agricultural Sciences, 2 Yuanmingyuan West Road, Beijing 100193, China; 2 International Agricultural Technology Information Institute, Hankyong National University, 327 Jungang Road, Anseong 17579, Republic of Korea

**Keywords:** Bcest, *Botrytis cinerea*, germination, hyphal growth, pathogenicity, stress tolerance

## Abstract

*Botrytis cinerea* is a necrotrophic model fungal plant pathogen that causes grey mould, a devastating disease responsible for large losses in the agriculture sector. As important targets of fungicides, membrane proteins are hot spots in the research and development of fungicide products. We previously found that membrane protein Bcest may be closely related to the pathogenicity of *Botrytis cinerea*. Herein, we further explored its function. We generated and characterised Δ*Bcest* deletion mutants of *B. cinerea* and constructed complemented strains. The Δ*Bcest* deletion mutants exhibited reduced conidia germination and germ tube elongation. The functional activity of Δ*Bcest* deletion mutants was investigated by reduced necrotic colonisation of *B. cinerea* on grapevine fruits and leaves. Targeted deletion of Bcest also blocked several phenotypic defects in aspects of mycelial growth, conidiation and virulence. All phenotypic defects were restored by targeted-gene complementation. The role of Bcest in pathogenicity was also supported by reverse-transcriptase real-time quantitative PCR results indicating that melanin synthesis gene *Bcpks*13 and virulence factor *Bccdc*14 were significantly downregulated in the early infection stage of the Δ*Bcest* strain. Taken together, these results suggest that Bcest plays important roles in the regulation of various cellular processes in *B. cinerea*.

## 1. Introduction

*Botrytis cinerea* Pers.: Fr. (teleomorph: *Botryotinia fuckeliana Whetzel*) is a typical necrotrophic ascomycete and worldwide plant pathogen that infects crop hosts during both pre- and post-harvesting phases [1,2]. Moreover, *B. cinerea* infects over 1400 plant species including many economically important crops, and leads to tremendous economic losses [3]. Due to the lack of resistant varieties, chemical control remains the most effective strategy for grey mould management [4]. Nevertheless, through genetic plasticity, *B. cinerea* has developed resistance to many types of fungicides. Researchers use *B. cinerea* as a model fungus in molecular studies; therefore, exploring the molecular mechanisms underlying the development and virulence of *B. cinerea* will contribute to establishing more effective disease control strategies.

Membrane proteins play key roles in the physiological processes of microorganisms, including transportation, intercellular communication and drug targets. Membrane proteins are recognised and inserted into the lipid bilayer by exquisite cellular machineries, such as the GlpG rhomboid protease, which is thought to allow docking of a transmembrane substrate [5]. However, transporters are integral membrane proteins with central roles in the efficient movement of molecules across biological membranes. The nucleobase ascorbate transporter UapA from *Aspergillus nidulans* must dimerise for correct trafficking to the membrane [6]. Many membrane proteins are primary drug targets, especially those involved in converting extracellular signals into intracellular processes. Among them, G protein-coupled receptors (GPCRs) are crucial for cellular responses to a range of bioactive molecules, and they play a key role in signalling, including increasing the basal activity of the cannabinoid 2 receptor [7]. Interestingly, cell membrane proteins are important targets of fungi in the prevention and control of fungal diseases by fungicides [8].For example, natamycin inhibits the growth of yeast and fungi by inhibiting plasma membrane transporters that regulate amino acid and glucose transport [9].

In our previous study, we identified and characterised membrane protein Bcest, which was associated with the pathogenicity of *B. cinerea* [10]. Through homologous protein searches, we found that homologs of Bcest included adhesins (Ata/Bmac), peptidoglycan DL_endopeptidase, ATP-binding cassette (ABC) transporters, EkdA and lipasesproteins, and others. Among them, adhesin Ata is an important virulence factor that can promote the formation of bacterial biofilms [11]. Adhesin BmaC facilitates attachment between bacteria and hosts [12]. Peptidoglycan DL_endopeptidase SadA causes cell aggregation and promotes biofilm formation [13]. The absence of transporter PltI affects the inability of strains to produce antibiotic Plt [14]. In the present study, we investigated the role of membrane protein Bcest in fungal growth and pathogenesis to bridge this gap in knowledge about *B. cinerea.*

In order to determine the role of Bcest in *B. cinerea*, we constructed and characterised ∆*Bcest* deletion mutants. Deletion of the *Bcest* gene not only led to reduced pathogenicity and lower conidiation, but also increased sensitivity to H_2_O_2_. These results indicate that Bcest is involved in several processes in *B. cinerea*, including vegetative differentiation, adaptation to oxidative stress and triadimefon, and virulence.

## 2. Materials and Methods

### 2.1. Fungal Strains and Culture Conditions

Strain B05.10 of *B. cinerea* Pers.: Fr. (*B. fuckeliana* (de Bary) Whetzel) was isolated from *Vitis vinifera* and has been widely used as a standard reference strain [15]. *B. cinerea* was grown on potato dextrose agar (PDA; 200 g potato, 20 g dextrose, 20 g agar, 1 L water), potato dextrose broth (PDB; 200 g potato, 20 g dextrose, 1 L water), a complete medium (CM; 1 g yeast extract, 0.5 g casein acid hydrolysate, 0.5 g hydrolysed casein, 10 g glucose, 4 mM Ca(NO_3_)_2_·4H_2_O, 1.5 mM KH_2_PO_4_, 1 mM MgSO_4_·7H_2_O, 2.5 mM NaCl, 20 g agar, 1 L water) and a minimal medium (MM; 10 mM K_2_HPO_4_, 10 mM KH_2_PO_4_, 4 mM (NH_4_)_2_SO_4_, 2.5 mM NaCl, 2 mM MgSO_4_, 0.45 mM CaCl_2_, 9 μM FeSO_4_, 10 mM glucose, 20 g agar, 1 L water, pH 6.9) [16,17].

Conidia were quantified after 10 days of incubation on the PDA medium, washed from plates, diluted to 5 mL with ddH_2_O, and counted with a hemocytometer under a light microscope (×40). Growth tests under different stress conditions were performed on PDA plates supplemented with different agents including H_2_O_2_ (10 mM), KCl (1 M) and sodium dodecyl sulphate (SDS; 0.02%) [18]. The percentage of mycelial radial growth inhibition (RGI) was calculated using the formula RGI = ([C − N]/[C − 5]) × 100, where C and N indicate the colony diameter of the control and treatments, respectively [18]. Each experiment was repeated three times.

### 2.2. Gene Deletion and Complementation

To replace the *Bcest* gene in wild-type (WT) strain B05.10, 1000 bp upstream and 1000 bp downstream flanking sequences of *Bcest* were amplified by PCR from the genomic DNA of B05.10. The resulting amplicons were fused with the HPH hygromycin resistance gene using double-joint PCR [19]. Protoplast preparation and transformation were performed as previously described [20]. The resulting hygromycin-resistant transformants were preliminarily screened by PCR with primers (Supplementary Table S1), and further confirmed by Southern blotting analysis. The upstream fragment of *Bcest* was used as a probe and labelled with digoxigenin (DIG) using High Prime DNA Labelling and Detection Starter Kit I (Roche Diagnostics, Mannheim, Germany) in accordance with the manufacturer’s protocol. Genomic DNA was digested with *EcoR*I endonuclease. For complementation assays, a *Bcest* green fluorescent protein (GFP) cassette was generated by amplifying the entire open reading frame (ORF) of *Bcest* (without a stop codon) and cloning it into the pNAN–OGG vector containing the GFP gene and the nourseothricin resistance gene [21]. The resulting construct was confirmed by DNA sequencing and transformed into the *Bcest* deletion mutant.

### 2.3. Transcriptome Analyses

The mycelia of WT B05.10 and *Bcest* gene deletion mutant Δ*Bcest* were harvested after growth on the PDA medium at 22 °C under 12 h of light and 12 h of darkness for 3 days (with three biological replicates). Total RNA was extracted using a fungal RNA kit (R6840-01; Omega Bio-Tek, Norcross, GA, USA), using Nanodrop (Thermo Scientific, Waltham, MA, USA) for quality testing and using Agilent 2100 Bioanalyzer (Agilgent, Santa Clara, CA, USA) for obtaining the RNA integrity number (RIN) [22]. RNA samples with a RIN of >7.0, a 260/280 ratio of >1.8, and a 260/230 ratio of >1.9 were analysed by Allwegene Technology Co., Ltd. (Beijing, China). Briefly, Trimmomatic (v0.33) software was used to filter the sequencing data [23]. A reference genome index was built and filtered reads were mapped to the reference genome using STAR (v2.5.2b). Mapping statistics are shown in Supplementary Table S2. HTSeq (v0.5.4) was used to compare the read count values for each gene with the original gene expression level, and the value of fragments per kilobase of exon per million mapped reads (FPKM) was used to standardise the expression. DESeq (v1.10.1) was used to analyse differentially expressed genes (DEGs) with an absolute log2 value of >1 and a *p* value of <0.05 as the cutoff criteria. All DEGs are listed in Supplementary Table S3. Gene ontology (GO) categories of upregulated and downregulated genes were identified using the g:Profiler toolset [24].

### 2.4. Nucleic Acid Manipulation and qRT-PCR

Fungal genomic DNA was extracted as described previously [25]. Plasmid DNA was isolated using RapidLyse Plasmid Mini Kit (DC211; Vazyme, Nanjing, China).

Real-time quantitative reverse-transcription PCR (qRT-PCR) was used to measure the expression of disease-related genes in the *Bcest* disruption mutant Δ*Bcest* and the WT B05.10 strain. The total RNA remaining from the transcriptome experiment was used. RNA was reverse-transcribed using HiScript III 1st Strand cDNA Synthesis Kit (R312; Vazyme, Nanjing, China). qRT-PCR was performed using Taq Pro Universal SYBR qPCR Master Mix (Q712; Vazyme, Nanjing, China) and using specific primers and a thermal program to determine the expression of *Bcactin* (reference gene), Bcin14g02370, Bcin10g01030 and other genes. The expression level of each transcript was calculated using the ΔΔC_t_ method [26]. For the normalisation of the data, the transcription level of each gene in the hyphae of B05.10 was given a value of 1.0, and the scale was used to calibrate the transcript levels of genes in the hyphae of Δ*Bcest*. qRT-PCR was repeated three times. All genes and primers used for qRT-PCR are listed in Supplementary Table S4.

### 2.5. Pathogenicity and Infection-Related Morphogenesis Assay

Infection tests were performed on grape fruits and leaves. Briefly, the tested plant tissues were point-inoculated with 5 mm diameter mycelial plugs of 3-day-old cultures. Before inoculation, the cuticles of hosts were wounded with a sterilised needle tip to facilitate the penetration of the fungus into plant tissues. The inoculated samples were placed under high relative humidity conditions (~95%) at 25 °C with 16 h of daylight. These experiments were repeated three times and each included 10 samples. Infection-related morphogenesis was observed on an onion epidermis using a published method [27].

### 2.6. Morphology and Ultrastructure of Fungal Hyphae

To investigate the role of Bcest on hypha morphology and ultrastructure in ∆*Bcest* and WT B05.10 strains, scanning electron microscopy (SEM) and transmission electron microscopy (TEM) were performed. The mycelial morphology and ultrastructure were observed by SEM/TEM in accordance with a modified method [28]. The hyphae on coverslips were immersed in 4 °C glutaraldehyde (4%) and incubated in darkness at 4 °C for 16 h. Mycelia were washed three times with phosphate-buffered saline (PBS), dehydrated, and dried in a vacuum freeze-dryer. Samples were sprayed with gold powder and examined with an SU8000 SEM instrument (Hitachi, Tokyo, Japan). One millilitre of spore suspension (1 × 10^5^ spores mL^−1^) was added to 100 mL of PDB and incubated at 25 °C with shaking a 150 rpm for 72 h. Mycelia were centrifuged, washed three times with PBS, and postfixed with 1% osmium tetroxide for 2 h. Samples were washed three more times with PBS, further dehydrated in a graded ethanol series (30%, 50%, 60%, 70%, 80%, 90%, 95% and 100%), then embedded in Spurr’s low-viscosity resin. Sections were observed using an H-7500 TEM instrument (Hitachi, Tokyo, Japan).

### 2.7. Abiotic Stress and Pathogenic Factor Assay

Mycelia-responsive trials were carried out to determine the responses of B05.10, Δ*Bcest*, and the complemented strain Δ*Bcest*-C to abiotic stresses, including osmotic pressure, H_2_O_2_, SDS, protease, polygalacturonase and cellulase, and their ability to produce an infectious agent. Specifically, mycelial agar plugs were removed from the margin area of a 2-day-old PDA culture of an isolate and inoculated in Petri dishes containing PDA with KCl (1 M), H_2_O_2_ (10 mM) and 20 mg/L of SDS (*w*/*v*). Cultures were incubated at 20 °C for 2 days. Secretions of proteases, polygalacturonases and cellulases were assessed using nutrient agar (NA; 50 g skimmed milk powder, 5 g NaCl, 10 g sucrose, 3 g beef extract, 3 g yeast extract, 20 g agar, 1 L water, pH 7.0), polygalacuronic acid agar (PGAA; 10 g polygalacuronic acid, 20 g sucrose, 2 g (NH_4_)_2_SO_4_, 20 g agar, 1 L water) and carboxymethyl cellulose sodium agar (CMC-Na; 10 g carboxymethyl cellulose sodium salt, 10 g yeast extract, 1 g tryptone, 4 g (NH_4_)_2_SO_4_, 2 g K_2_HPO_4_, 0.5 g MgSO_4_·7H_2_O, 20 g agar, 1 L water) mediums, respectively. Cultures were incubated at 22 °C for 3 days. Experiments included one mycelial agar plug per dish and three dishes (replicates) for each treatment. The diameter of each colony was measured, and the mycelial growth inhibition rate (MGIR) was calculated using the following formula [29]:

MGIR = (AD_CK_ − Ds)/AD_CK_ × 100%.

where AD_CK_ is the average colony diameter of an investigated isolate in the control treatment, and Ds is the diameter of that isolate in the presence of a stress generation chemical (KCl, H_2_O_2_ or SDS). Each assay was repeated three times.

### 2.8. Statistical Analyses

All assays were conducted in triplicate, unless otherwise indicated. Conidia number, colony diameter and lesion diameter analyses were performed using IBM SPSS statistics 20.0 software (IBM Corp., Armonk, NY, USA). The significance of the effects of different treatments on various indices was evaluated with an analysis of variance (ANOVA) with multiple least-significant-difference comparisons at the *p* ≤ 0.05 level. After analysis, average angular values were individually back-transformed to numerical values.

## 3. Results

### 3.1. Identification of Bcest in B. cinerea

**Figure 1:**
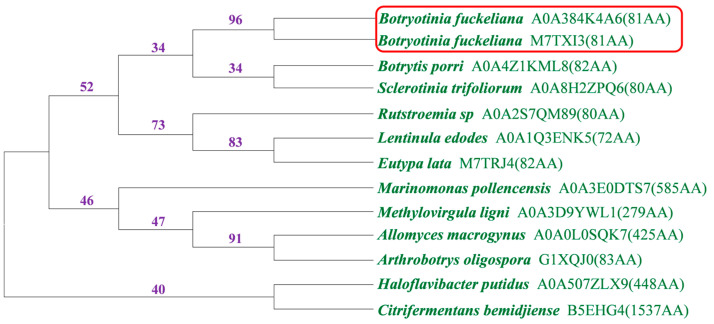
Phylogenetic tree of Bcest proteins based on a neighbour-joining analysis using MEGA. Numbers represent boostrap values.

The *Bcest* gene (Bcin15g00520) of B. cinerea was identified via transcriptome data analysis. Bioinformatic analysis showed that this 874 bp gene with three exons and two introns encodes an 81-amino acid protein. Homologs of Bcest were identified via BLASTp and phylogenetic trees of Bcest proteins were constructed vis MEGA 10.0.5 (Figure 1). Evolutionary history was inferred using the neighbour-joining method with 1000 bootstrap replications [30]. It can be seen from Figure 1 that the Bcest protein A0A384K4A6 is highly homologous to putative EkdA protein M7TXI3 from the *B. cinerea* BcDW1 strain.

### 3.2. Deletion and Complementation of Bcest in B. cinerea

**Figure 2:**
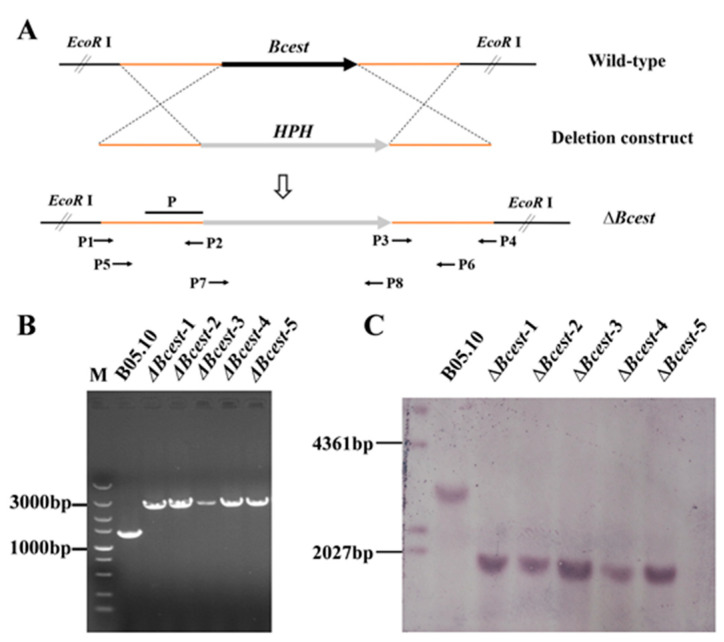
Target gene deletion. (**A**) Schematic diagram of the *Bcest* homologous replacement strategy. (**B**) Amplification of *Bcest* recombinant fragments in B05.10 and *Bcest* gene deletion mutants. (**C**) Southern blotting of *Bcest* gene deletion mutant strains.

To investigate the functions of the Bcest protein in *B. cinerea*, we generated single-gene deletion mutants of Δ*Bcest* using homologous recombination (Figure 2A). The left and right arms (1000 bp) of the *Bcest* gene and the hygromycin gene (2145 bp) of plasmid pUCHYG were amplified. The recombinant *Bcest* gene containing the above fragments was obtained via fusion PCR.

We obtained independent transformants via screening a selection medium supplemented with hygromycin B and PCR verification. After single0spore isolation, transformants were verified as homozygous via PCR and further confirmed to be single-copy insertions via Southern blotting analysis (Figure 2B,C). To confirm that the phenotypic changes of the mutants were due to gene deletion, Δ*Bcest* mutants were complemented with the full-length *Bcest* gene to generate complemented strains of Δ*Bcest*-C.

### 3.3. Bcest Is Involved in Hyphal Growth and Conidiation

**Figure 3:**
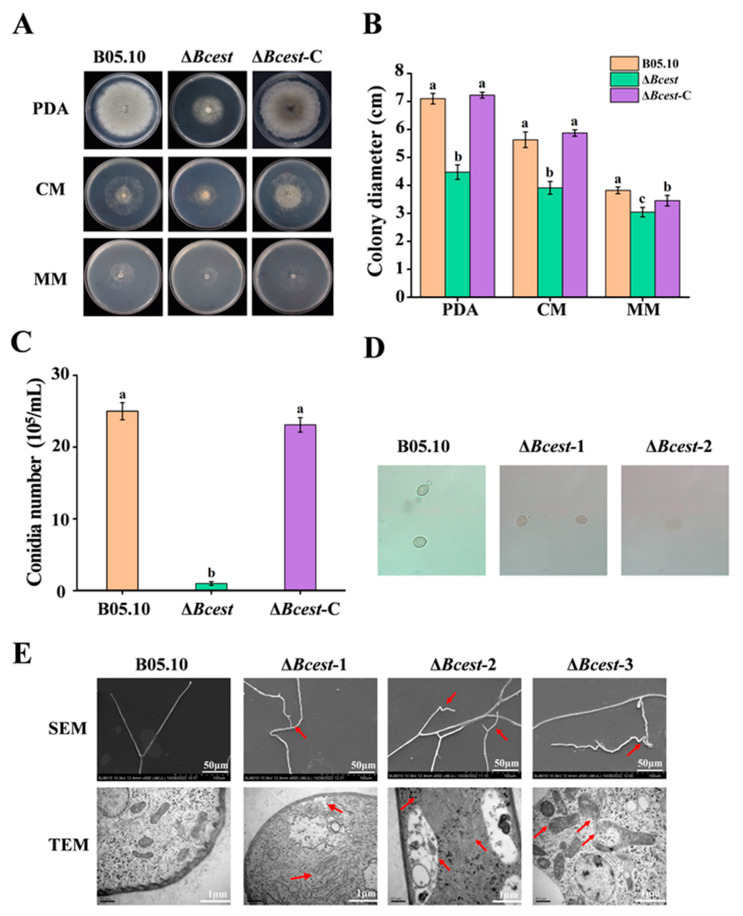
Effects of Bcest deletion on mycelial growth, sporulation and conidial germination. Bars represent standard errors from three replicates. Values on bars followed by different letters indicate significant differences at *p* = 0.05. (**A**) Mycelial growth of ∆*Bcest*, B05.10 and ∆*Bcest*-C strains on PDA plates after 3 days of cultivation. (**B**) Quantification of colony diameter of the indicated strains grown on PDA plates for 3 days. (**C**) Quantification of conidia produced by the indicated strains. (**D**) Conidia morphology of different strains. (**E**) SEM and TEM observations of hyphae of *B. cinerea* and ∆*Bcest* strains grown on PDA plates (diameter: 4 cm). Note: The red arrows in TEM highlight abnormal hyphal growth and uneven branching, and in TEM highlight cell membrane invaginations, mitochondrial swelling, and mitochondrial membrane disappearance.

The mycelial growth rate and the conidium of Δ*Bcest* were significantly different from the those of the WT parent B05.10. The Δ*Bcest* strain had a slower growth rate than the Δ*Bcest*-C complemented strains and the B05.10 WT strain did on PDA, CM and MM, but especially on PDA (Figure 3A,B). Conidia are the primary inoculum for the disease cycle of *B. cinerea* [31]. After incubating it on PDA for 10 days, the abundance of conidia for Δ*Bcest* was significantly less than that for B05.10 and Δ*Bcest*-C (Figure 3C). Interestingly, the conidia of Δ*Bcest* showed morphological and size abnormalities, and some spores produced by Δ*Bcest* had folds and cracks on the surface (Figure 3D). In addition, when incubated on the PDA medium at 22 °C for 10 h, all spores of B05.10 germinated, whereas the average germination rate of Δ*Bcest* was only 78.03%.

SEM results showed that control strain B05.10 hyphae grew upright, with uniform thickness, a smooth surface, and no distortion or deformities, and apical hyphae branches were relatively uniform; by contrast, those of Δ*Bcest* showed distortion and deformities, with surface folds and depressions, differences in thickness, and uneven apical branches. TEM results showed that compared with control strain B05.10, the amount of endoplasmic reticulum and the number of mitochondria were increased in Δ*Bcest*, the mitochondrial membrane had disappeared at one end, and mitochondria were larger, looser and more folded. Furthermore, cell membranes were relatively intact, but tended to sink inwards. The above features are indicated by red arrows in Figure 3E.

These results also indicate that Bcest is important for vegetative growth and the conidiation of *B. cinerea*.

### 3.4. Bcest Participates in Regulating the Pathogenicity of B. cinerea

**Figure 4:**
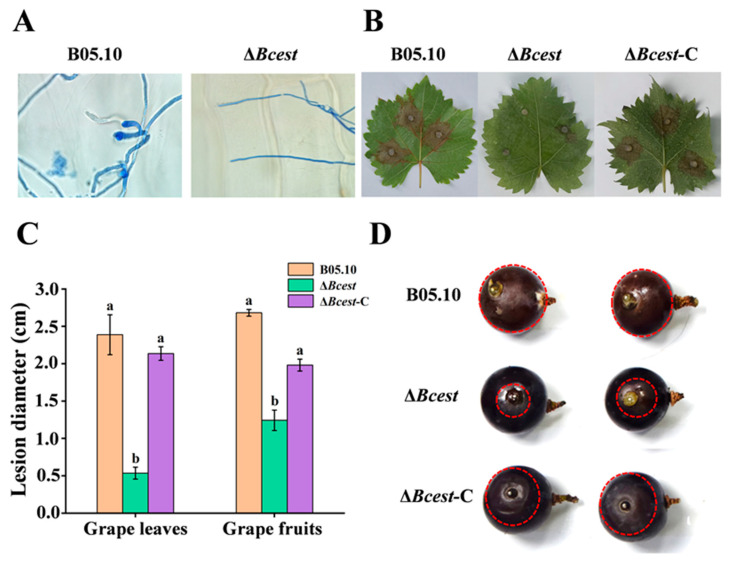
Effects of Δ*Bcest* deletion on mycelial infection and pathogenicity. Bars represent standard errors from three replicates. Values on bars followed by different letters indicate significant differences at *p* = 0.05. (**A**) Onion epidermis penetration by B05.10 and Δ*Bcest*. Pictures were taken after 12 h of inoculation of onion epidermis with conidia from B05.10 and Δ*Bcest*. (**B**) Disease symptoms caused by each strain on wounded grape leaves and fruits. Images were captured at 96 h after inoculation. (**C**) Pathogenicity on grape leaves after 96 h of incubation. (**D**) Pathogenicity on grape fruits after 96 h of incubation on PDA plates (diameter 4 cm). Note: we used Δ*Bcest*-1, Δ*Bcest*-2 and Δ*Bcest*-3 strains for the pathogenicity test of the Δ*Bcest* strain. The red circle represents the diameter of the lesion.

To determine and visually observe whether or not Bcest is involved in regulating the infection capacity of *B. cinerea*, an onion epidermis was inoculated with the Δ*Bcest* mutant spore suspension. At 12 h, unlike WT spores, Δ*Bcest* spores failed to form an infection structure after germination, and the WT strain successfully infected the onion epidermis by generating attachment cells after spore germination (Figure 4A).

To determine whether or not Bcest is involved in regulating pathogenicity in *B. cinerea*, grapevine leaves and fruits were inoculated with Δ*Bcest* mutants. Compared with the WT strain, Δ*Bcest* mutants exhibited reduced virulence in different hosts (Figure 4B). At 96 h, grape leaves inoculated with Δ*Bcest* mutants showed no or small lesions, while WT-inoculated leaves showed larger lesions and the average lesion size reached 0.53 cm and 2.13 cm, respectively (Figure 4C). Similarly, lesion size was considerably decreased on Δ*Bcest* mutant-inoculated grape fruits compared with WT-inoculated fruits (Figure 4D). The complemented strain Δ*Bcest*-C exhibited almost the same level of virulence as the WT strain did. These results suggest that Bcest plays a crucial role in virulence of *B. cinerea*.

### 3.5. Effects of ΔBcest Deletion on Sensitivity to Abiotic Stresses and Pathogenicity Factors

**Figure 5:**
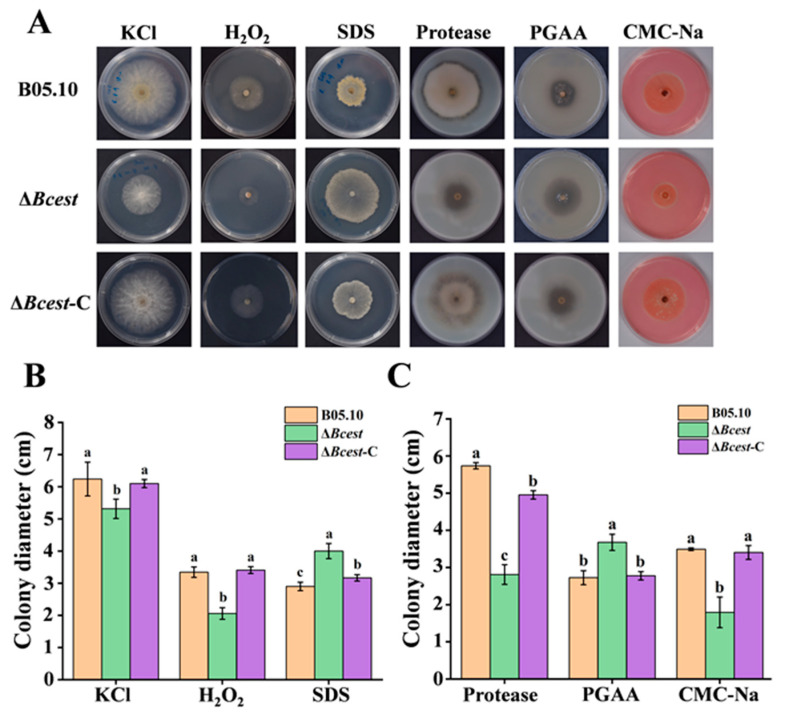
Detection of sensitivity to abiotic stress and the ability to secrete virulence factors in the Δ*Bcest*, B05.10, and Δ*Bcest*-C strains. (**A**) All strains were grown on PDA plates amended with NaCl, KCl, or SDS at the indicated concentrations at 20 °C for 2 days, and with skimmed milk powder, polygalacuronic acid or carboxymethyl cellulose at 20 °C for 3 days. (**B**) Sensitivity of Δ*Bcest*, B05.10 and Δ*Bcest*-C to KCl, H_2_O_2_ and SDS. (**C**) Ability of Δ*Bcest*, B05.10 and Δ*Bcest*-C to produce proteases, polygalacturonase and cellulases. Bars represent standard errors from three replicates. Values on bars followed by different letters indicate significant differences at *p* = 0.05.

The results of the mycelial responsive assays showed that compared with Δ*Bcest*-C and B05.10, Δ*Bcest* mutants exhibited suppressed mycelial growth in the presence of KCl and H_2_O_2_, and the transparent zone in the protease and cellulase test medium was significantly reduced. However, the growth diameter under the SDS treatment and the polygalacturonase production capacity of Δ*Bcest* were significantly higher than those of B05.10 and Δ*Bcest*-C, respectively (Figure 5A). The values were not significantly different (*p* = 0.05) from the inhibition rates for B05.10 and Δ*Bcest*-C in response to these chemicals, and the enzyme-producing capacity was not significantly different either (Figure 5B,C). These results suggest that disruption of Bcest may have marginal effects on mycelial growth in response to abiotic stresses, and that pathogenicity factor production capacity may also be affected.

### 3.6. Bcest Deletion Affects Transcription and Pathogenicity-Related Genes

**Figure 6:**
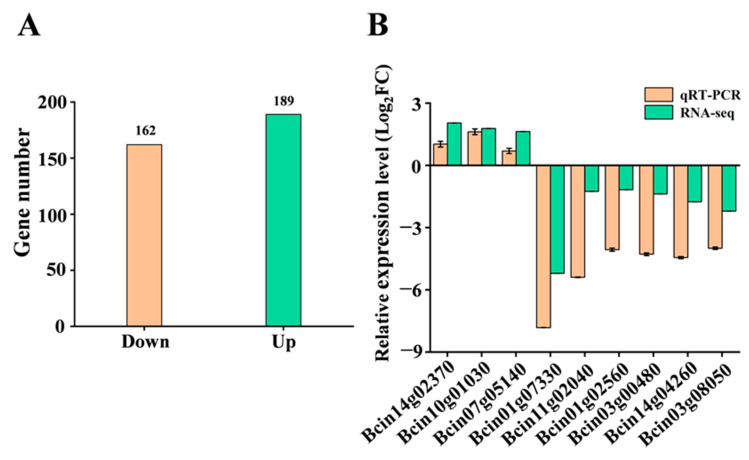
RNA-seq analysis of Δ*Bcest* deletion strains. (**A**) Numbers of upregulated and downregulated genes (*p* < 0.05, >2-fold change) in Δ*Bcest* strains compared with those in WT B05.10. (**B**) qRT-PCR of Δ*Bcest* transcriptome DEGs.

We performed a RNA-seq analysis to identify genes that might exhibit changes in regulation affected by Bcest in *B. cinerea*. Three biological replicates with mRNA isolated from WT B05.10 and Δ*Bcest* strains were performed, and 162 downregulated and 189 upregulated (fold change > 2, *p* < 0.05) genes were identified in Δ*Bcest* and compared with those of B05.10 (Figure 6A).

Functional annotation of DEGs via GO analysis was performed to identify genes belonging to molecular function, cellular component and biological process categories (Figure S1). Among them, catalytic activity/oxidoreductase activity, organelle envelope/mitochondrial outer membrane, the oxidation–reduction process and the monocarboxylic acid metabolic process were the main molecular function, cellular composition, and biological process subcategories, respectively. These results showed that growth and pathogenicity defects caused by the absence of Bcest in the B05.10 strain may be closely related to these six terms.

In addition, enriched DEGs can be functionally classified into metabolism, cellular processes and genetic information processing categories. Among the 45 metabolic pathways belonging to these three categories, the top 10 metabolic pathways of enriched DEGs were metabolic pathways, biosynthesis of secondary metabolites, biosynthesis of antibiotics, carbon metabolism, biosynthesis of amino acids, oxidative phosphorylation, glutathione metabolism, tricarboxylic acid (TCA)/citrate cycle, the pentose phosphate pathway, and ubiquitin–mediated proteolysis (Figure S2). These metabolic pathways may be closely related to the growth and pathogenicity defects of Δ*Bcest*.

To verify the reliability of DEGs identified from transcriptome sequencing, qRT-PCR was performed on the remaining Δ*Bcest* RNA samples used for transcriptome analysis. qRT-PCR validation was performed by randomly selecting nine growth- and pathogenicity-associated genes. These genes encode proteins that participate in the synthesis and metabolism of substances (Bcin14g02370, Bcin07g05140, Bcin03g08050, and Bcin11g02040), the catalytic reactions of enzymes (Bcin03g00480, Bcin01g07330, and Bcin10g01030) and virulence and stress factors (Bcin14g04260 and Bcin01g02560). The qRT-PCR results were consistent with the RNA-seq results (Figure 6B), indicating the reliability of the RNA-seq data in this study.

## 4. Discussion

To explore the functions of membrane protein Bcest, we first disrupted the *Bcest* gene and characterised the resulting mutant, which showed severe defects in hyphal growth and pathogenicity. These results are consistent with those of experiments on *BcHBF1* and *BcATG6* genes of *B. cinerea* [32,33]. We thus hypothesised that the Bcest protein might be involved in regulating hyphal growth and pathogenicity-related genes in this fungal species. Bcest is a vital virulence determinant, since deletion of the *Bcest* gene also compromised the penetration ability of *B. cinerea*, indicating that the reduced virulence of the Δ*Bcest* mutant was likely due, as least in part, to defects in the penetration of host cells. The Δ*Bcest* mutant exhibited increased sensitivity to H_2_O_2_ and osmotic stress, which may be due to the absence of membrane protein Bcest, which destroys mitochondrial membrane integrity, reducing tolerance to osmotic pressure and reactive oxygen species (ROS) [34]. However, under SDS-mediated stress, the growth rate of the Δ*Bcest* mutant strain was higher than that of WT strain B05.10. However, other studies have shown that SDS can inhibit the growth of *B. cinerea* [33]; hence, the specific mode of action will be explored in our future research. In addition, the ability of the Δ*Bcest* mutant strain to produce cellulases and proteases was significantly reduced, which is another important factor of pathogenicity [35,36]. Based on the above results, the Bcest protein appears to be an important virulence factor of *B. cinerea*. However, the regulatory mechanisms involving Bcest remain poorly understood, and further research such as a comparative analysis of transcription profiles could provide valuable information.

We used transcriptome sequencing technology to compare and analyse transcriptional regulation differences and DEGs of ∆*Bcest* mutant strains in culture for 72 h, and 351 DEGs were screened. GO functional analysis showed that in biological process and molecular function categories, DEGs resulting from the loss of the *Bcest* gene were mostly involved in metabolic and cellular processes, and catalytic activity and binding, respectively. Among the cellular component terms, the most enriched ones were linked to cellular structure and membrane, which is consistent with the fact that the deletion of the *Bcest* gene affected the mitochondrial membrane in the TEM experiment. Our RNA-seq analysis results suggest that global changes in genes involved in metabolic pathways, the biosynthesis of secondary metabolites, the pentose phosphate pathway and the TCA cycle are likely to underlie this defect.

The reliability of the transcriptome data was verified by via qRT-PCR of nine growth- and pathogenicity-related genes. Sclerotia are an important virulence factor in *B. cinerea*, and this study found that the melanin synthesis gene *Bcpks13* (Bcin03g08050) was significantly downregulated in the ∆*Bcest* mutant strain, which may explain why this strain failed to form sclerotia [37]. Deletion of *CDC14* in several plant pathogen species severely impairs virulence, demonstrating that *CDC14* function is important for host infection [38]. *Fusarium graminearum, Magnaporthe oryzae* and *Aspergillus flavus* lacking *CDC14* gene expression can lead to conidia formation defects and reduced pathogenicity [39,40,41]. Polygalacturonase and β-1,4-endoxylanase *BcXyn11A* are important virulence factors of *B. cinerea*, and they mainly promote the virulence of *B. cinerea* through necrotic activity rather than enzymatic activity [42]. We found that *BcXyn11a* (Bcin03g00480), *Bccdc14* (Bcin01g02560) and endopolygalacturonase-encoding gene *Bcpg5* (Bcin01g07330) were significantly downregulated, which suggests that the decrease in the number of conidia and weakening of the pathogenicity of ∆*Bcest* mutant strains were the result of a combination of polygenes. *Bcgas2* (Bcin14g04260) is required for *B. cinerea* to cope with stress [43,44], and a decrease in the stress capacity of the ∆*Bcest* mutants was closely related to the downregulation of this gene. In addition, both the mitochondrial membrane and the mitochondrial electron transport chain can induce a burst of ROS, which in turn affects the growth and development of the strain. In this study, the expression of the cytochrome C synthesis gene *Bccyc1* (Bcin14g02370), which is involved in the mitochondrial electron transport chain, was significantly upregulated in ∆*Bcest* mutant strains [45]. Interestingly, we found that the membrane protein Bcest had little effect on cell membranes, but TEM showed that the mitochondrial membrane of the Δ*Bcest* strain was damaged, enlarged, wrinkled and relaxed. Thus, we hypothesised that loss of Bcest may destroy the integrity of the mitochondrial membrane, affect the transmission of the mitochondrial electron chain, and influence metabolic pathways in the strain. Therefore, we concluded that an absence of Bcest can reduce the growth rate and pathogenicity of *B. cinerea*, while increasing sensitivity to environmental stress.

## 5. Conclusions

In conclusion, membrane protein Bcest can inhibit the growth of *B. cinerea*, reduce the spore germination rate and pathogenicity, disrupt the integrity of the mitochondrial structure, and increase sensitivity to oxidative and osmotic stress.

## Data Availability

The data used in the study analyses can be made available by the corresponding author upon reasonable request.

## Supplementary Materials

The following supporting information can be downloaded at https://www.mdpi.com/article/10.3390/microorganisms11051225/s1. Figure S1: GO functional classification of Δ*Bcest* transcriptome DEGs. Figure S2: KEGG enrichment analysis bubble diagram of the Δ*Bcest* transcriptome. Table S1: Mapping statistics in transcriptome analyses. Table S2: Oligonucleotide primers used in this study. Table S3: List of all DEGs in transcriptome analyses. Table S4: Genes and primers used for qRT-PCR.
