# Supplementary material for: Membrane Protein Bcest Is Involved in Hyphal Growth, Virulence and Stress Tolerance of Botrytis cinerea
Source: Microorganisms. 2023 May 6;11(5):1225. doi: 10.3390/microorganisms11051225 (PMC10221684; doi:10.3390/microorganisms11051225)
Supplement: Supplementary file 1 [file microorganisms-11-01225-s001.zip › Table S1 Oligonucleotide primers used in this study.pdf]

Table S1. Oligonucleotide primers used in this study.

Primer	Sequence(5'-3')	Relevant characteristics
P1	AGGTAACGCCTATTACGTTTCAG	PCR primers to amplify *Bcest* upstream fragment for construction of the gene deletion vector
P2	ATATCATCTTCTGTCGACCTGCAGGCCACCAACTAATGCAAACCATCTA	
P3	TCTTTCTAGAGGATCCCCGGGTACCGAGCAACATAACCCAACAATGATG	PCR primers to amplify *Bcest* downstream fragment for construction of the gene deletion vector
P4	ATACCGGATTGAGGGATATTGC	
P5	AGGTAACGCCTATTACGTTTCAG	PCR primers to amplify the deletion vector of *Bcest* with the double-joint PCR products as template
P6	ATACCGGATTGAGGGATATTGC	
P7	CGGTACCCGGGGATCCTCTAG	PCR primers to amplify the hygromycin B (*HPH*) gene
P8	GCCTGCAGGTCGACAGAAGATG	
P9	GCCAATAAAGAAAGAAGAAAAA	PCR primers to identificate the *Bcest* deletion mutant
P10	CATTTAGAAAAGACTTCACAACC	
P11	ACAGGACACTTCTTTTCAGGC	PCR primers to amplify the probe fragment for *Bcest* Southern blotting
P12	TGTACAGTAGCATTCCGGATATT	
