# Supplementary figures and images for: Membrane Protein Bcest Is Involved in Hyphal Growth, Virulence and Stress Tolerance of Botrytis cinerea
Source: Microorganisms. 2023 May 6;11(5):1225. doi: 10.3390/microorganisms11051225 (PMC10221684; doi:10.3390/microorganisms11051225)

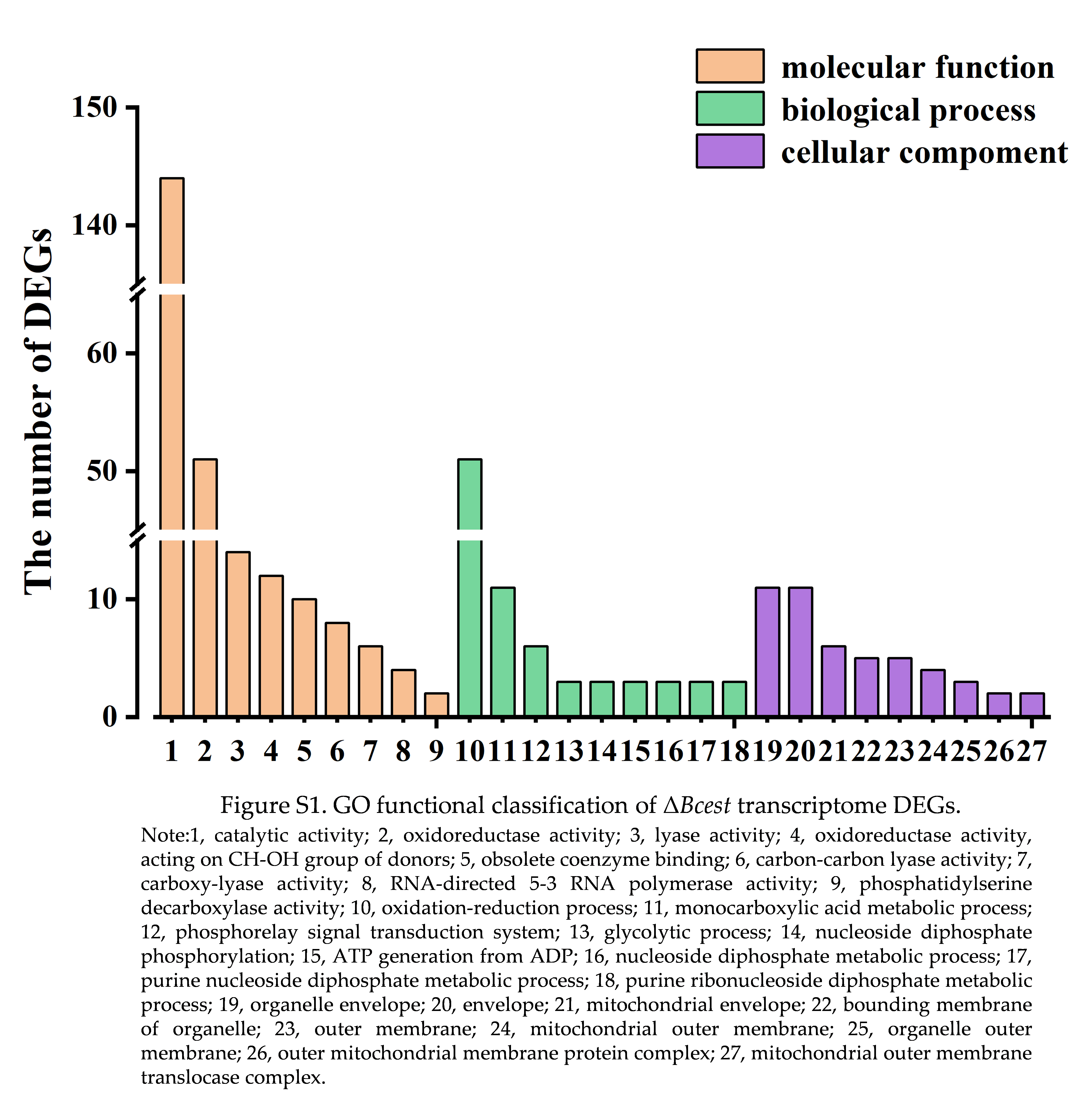

Supplement: Supplementary file 1 [file microorganisms-11-01225-s001.zip › Figure S1-GO functional classification of añBcest transcriptome DEGs.tif]

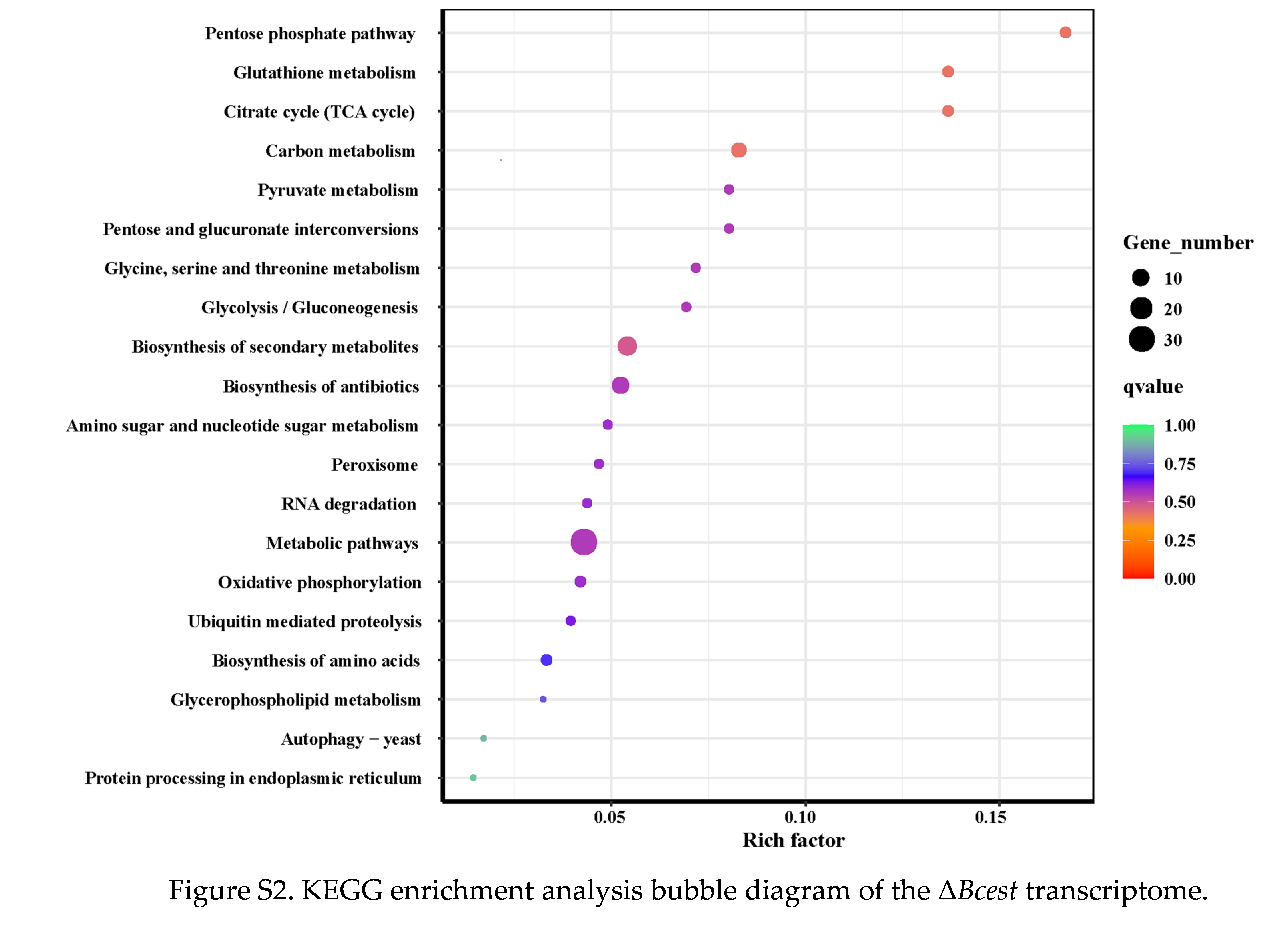

Supplement: Supplementary file 1 [file microorganisms-11-01225-s001.zip › Figure S2-KEGG enrichment analysis bubble diagram of the añBcest transcriptome.tif]
